# Transcatheter Aortic Valve Implantation: Addressing the Subsequent Risk of Permanent Pacemaker Implantation

**DOI:** 10.3390/jcdd10060230

**Published:** 2023-05-24

**Authors:** Philipp Lauten, Lisa C. Costello-Boerrigter, Björn Goebel, David Gonzalez-Lopez, Matthias Schreiber, Thomas Kuntze, Mahmoud Al Jassem, Harald Lapp

**Affiliations:** Department of Cardiology, Heart Center, Zentralklinik Bad Berka, Robert-Koch-Allee 9, 99437 Bad Berka, Germanybjoern.goebel@zentralklinik.de (B.G.); harald.lapp@zentralklinik.de (H.L.)

**Keywords:** TAVI, pacemaker, conduction disturbances, implantation depth, PPI

## Abstract

Transcatheter aortic valve implantation (TAVI) is now a commonly used therapy in patients with severe aortic stenosis, even in those patients at low surgical risk. The indications for TAVI have broadened as the therapy has proven to be safe and effective. Most challenges associated with TAVI after its initial introduction have been impressively reduced; however, the possible need for post-TAVI permanent pacemaker implantation (PPI) secondary to conduction disturbances continues to be on the radar. Conduction abnormalities post-TAVI are always of concern given that the aortic valve lies in close proximity to critical components of the cardiac conduction system. This review will present a summary of noteworthy pre-and post-procedural conduction blocks, the best use of telemetry and ambulatory device monitoring to avoid unnecessary PPI or to recognize the need for late PPI due to delayed high-grade conduction blocks, predictors to identify those patients at greatest risk of requiring PPI, important CT measurements and considerations to optimize TAVI planning, and the utility of the MInimizing Depth According to the membranous Septum (MIDAS) technique and the cusp-overlap technique. It is stressed that careful membranous septal (MS) length measurement by MDCT during pre-TAVI planning is necessary to establish the optimal implantation depth before the procedure to reduce the risk of compression of the MS and consequent damage to the cardiac conduction system.

## 1. Introduction

Given both the location of the aortic valve in the heart and the anatomical course of the cardiac conduction system, it is unsurprising that manipulations of the aortic valve can result in disturbances of the conduction system. This well-recognized complication after transcatheter aortic valve implantation (TAVI) in patients with aortic stenosis is of concern because post-TAVI permanent pacemaker implantation (PPI) has not decreased with time as much as would be desired, although other TAVI complications have become impressively less frequent as the procedure has become more common [1,2,3,4]. This apparent lack of progress in protecting the cardiac conduction system stems from the challenging anatomy in the region of the aortic valve. The atrioventricular node is typically located near the membranous septum (MS) and then continues as the bundle of His, which pierces the MS. Likewise, the origins of the coronary arteries are also in the aortic valvular region, usually just below the sinotubular junction [5]. Thus, operators are sometimes in the position of trying to avoid both coronary ischemia due to obstruction of the vessels and damage to the cardiac conduction system [5]. Ultimately, it is sometimes easier to “rescue” cardiac conduction by PPI; therefore, when the risk to the conduction system must be weighed against the risk of blocking a coronary artery, the coronary artery wins.

Over the course of the past 20 years, TAVI has proven to be a safe and effective treatment for aortic stenosis and so has expanded from being reserved as a treatment for patients at high or prohibitive risk of cardiac surgery to patients at intermediate or lower surgical risk [6,7,8]. In light of this, the risk of post-TAVI PPI also needs to be considered more as right ventricular pacing is not entirely benign. Importantly, right ventricular apical pacing causes dyssynchronous activation of the ventricles, which can ultimately cause remodeling of the left ventricle and heart failure and which, in turn, potentially increases the risk of death [9]. In the setting of right ventricular pacing, there is also an associated increased risk of tricuspid regurgitation and of atrial fibrillation, which carries along with it increased morbidity and mortality [10]. Physiological control of the heart rate is also gone [11]. Of note, in contrast to this, data from the SWEDEHEART registry suggests that the long-term survival of patients who underwent PPI after TAVI was no different from TAVI patients who did not undergo PPI [12]. Nevertheless, avoidance of PPI whenever possible is certainly a goal that is probably not being achieved [13,14]. The PARTNER Trial and Registry found that the majority of PPI (97.1%) occurred during the index hospitalization, generally within 7 days of the TAVI procedures [15]. It is noteworthy that conduction disturbances can at times be transient or “dynamic” in behavior, with some blocks disappearing with time [16]. In such cases, it would be undesirable to rush to PPI. Unnecessary, early PPI for transient or insignificant blocks is to be avoided, but persistent high-grade blocks need to be addressed. Also critical is the anticipation of delayed, life-threatening conduction blocks that would not become evident until after hospital discharge [17,18]. Thus, with conduction blocks after TAVI, a safe balance must be achieved. To achieve this balance, one must identify unchangeable patient predictors (anatomy and baseline EKG), procedural techniques and valve choice, and the type and extent of monitoring post-TAVI (prior to discharge and potentially after discharge)

Here, we will review the causes and types of conduction disturbances that are seen post-TAVI, pre-procedural, and peri-procedural predictive factors for PPI, techniques to help avoid such conduction disturbances, and new research directed at decreasing PPI. 

## 2. Cardiac Anatomy Lends Itself to Post-TAVI Conduction Blocks

**Figure 1:**
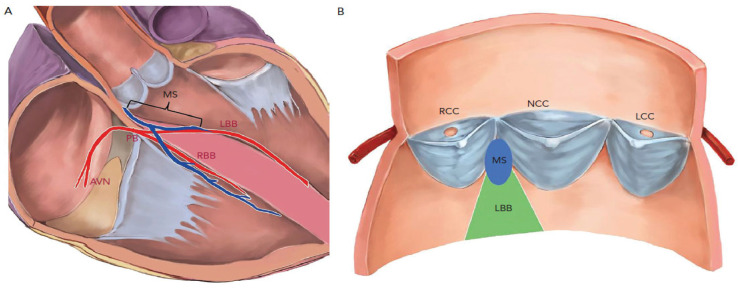
Figure from [25]. Anatomical Relationships Between the Aortic Cuspids, Membrane Septum and Conduction System. (**A**) The penetrating bundle of His emerges at the surface of the left ventricular outflow tract beneath the membrane septum (MS). The length of the MS is equal to the distance between the aortic annulus and bundle of His. (**B**) The left bundle branch emerges beneath the MS and is positioned between the right coronary cusp and non-coronary cusp. AVN = atrioventricular node; LBB = left bundle branch; LCC = left coronary cusp; PB = penetrating bundle; MS = membrane septum; NCC = non-coronary cusp; RBB = right bundle branch; RCC = right coronary cusp.

Conduction blocks are well-known complications of both TAVI and surgical aortic valve replacement [19,20]. That such procedures would cause injury to the conduction system is unsurprising given that the aortic valve lies in close proximity to fundamental components of the cardiac conduction system. The atrioventricular node is located near the apex of the Triangle of Koch, which thus means that it is close to the subaortic region and the MS [5]. The atrioventricular node gives rise to the bundle of His and pierces the MS and travels leftward through the central fibrous body, which is where the MS, the atrioventricular valves, and the aortic valve are all continuous with each other [21]. The left bundle branch of the bundle of His then exits just below the MS at the base of the “interleaflet triangle” that separates the aortic valve’s non-coronary and the right coronary leaflets [5]. Thus, manipulations of the aortic valve and implantation of a prosthetic valve can easily damage critical components of the cardiac conduction system. (Figure 1) Furthermore, certain anatomic variations of the atrioventricular node and bundle put some patients at increased risk of disruption of the conduction system. For example, in those cases where the bundle travels under the MS or where the bundle is more on the left, then there is a greater risk of injury to the conduction system than when the bundle is more towards the right side. This risk is even higher if the patient also has a short MS [22,23]. Additionally, a narrow left ventricular outflow tract (LVOT) diameter or calcification, particularly below the non-coronary cusp (NCC), make compression of the MS (and potentially then the left bundle) by the prosthetic valve more likely [4,24]. 

## 3. Most Common Conduction Blocks Post-TAVI and Subsequent Need for PPI

### 3.1. Left Bundle Branch Block (LBBB) 

The incidence of new-onset, post-TAVI LBBB is high, although the range is broad (5–65%), and, this incidence is only slightly decreasing with more modern prostheses [21,26,27]. It is worth noting that a new LBBB, which typically evolves during the intervention and only rarely develops more than a month later, is also often transient, resolving even before hospital discharge. However, if the LBBB persists beyond 30 days post-discharge, then it is most likely to be permanent [28,29,30,31,32,33]. It has been proposed that, in patients with pre-procedural right bundle branch block (RBBB), iatrogenic injury by a stent or balloon in the area of the LVOT or aortic annulus is of particular concern as a new LBBB could result [17]. The combined pre-existing RBBB and the new LBBB produce a situation in which PPI is extremely likely and so close monitoring is required [17].

### 3.2. Atrioventricular Block (AVB) 

The incidence of complete (third-degree) AVB after TAVI is approximately 20%, and complete or high-grade AVB (Mobitz I or Mobitz II second-degree AVB) represents the primary reason for PPI after TAVI [15,34,35]. Due to procedure-related trauma, high-grade AVB frequently occurs immediately after valve deployment or peri-procedurally and often can resolve on its own, although temporary pacing during the procedure and for 1–2 days post-TAVI may be needed [36,37]. However, complete or high-grade AVB might not be observed to resolve, and often PPI occurs during the same hospitalization as the TAVI, with a reported median time to PPI of 3 days [38]. Furthermore, sometimes late/delayed high-grade AVB has been known to occur, and this could even result in syncope or sudden cardiac death after the patient has been discharged from the hospital [39]. In keeping with this, it has been noted that post-TAVI patients who have a persistent, new LBBB, QRS >150 ms, and PR interval >240 ms are at greater risk of going on to develop a delayed high-grade AVB that requires PPI [40,41]. Likewise, patients with atrial fibrillation who develop post-TAVI bradycardia with a ventricular rate <50 bpm are generally considered to have high-grade AVB requiring PPI [18,42].

## 4. Possible Pre-Procedural Predictors for PPI

Prior to performing a TAVI procedure, there are some patient-specific factors that need to be considered as they may increase a patient’s risk of needing PPI.

### 4.1. Demographic Predictors

#### 4.1.1. Male Sex

Although data from small studies have been conflicting, the summary effect from meta-analyses supports the concept of a sex-dependent risk of PPI after TAVI, with males at greater risk of PPI. Although women have higher in-hospital mortality rates and more vascular complications, men are considered to be statistically more likely to require PPI [35,43,44]. This may be secondary to the use of larger bioprostheses in males or to their other comorbidities [43]. However, a recent report using retrospectively analyzed data from the Netherlands Heart Registration, a very large national database, suggests that the male sex protects against requiring post-TAVI PPI. This may be because men, in general, have a larger aortic annulus and thus oversizing occurs less frequently [45]. How one is to reconcile these conflicting results remains unclear, but in the interim one could perhaps consider male sex to be at least a weak pre-procedural predictor for PPI.

#### 4.1.2. Age

The role of age as a predictor of PPI is not clear-cut in some meta-analyses; however, large national registries, such as recent reports from France and Switzerland, have found increasing age was associated with an increased risk of PPI [13,43,46]. Likewise, a substudy of the large, multi-national PRAGMATIC registry found that age was predictive of PPI (OR 1.08, 95% CI 1.04−1.12, *p* < 0.0001) [47].

#### 4.1.3. Other Factors?—Body Mass Index (BMI), Serum Creatinine

Other patient-related factors that may be predictive, although studies have yielded inconsistent results, include BMI and serum creatinine. Both the substudy of the Pragmatic registry (OR 1.08, 95% CI 1.02–1.13, *p* < 0.004) and the Netherlands Heart Registration data suggest that, rather than seeing a protective “obesity paradox”, that increased BMI was associated with an increased risk of PPI [45,47]. Likewise, impaired renal function has been reported to be predictive of post-TAVI PPI in the Netherlands Heart Registration (OR 1.15, 95% CI 1.01–1.31, *p* < 0.04) and in a French national hospital database (OR 1.09, 95% CI 1.02–1.17, *p* < 0.01) [13,45].

### 4.2. EKG and CT Predictors

#### 4.2.1. Short Membranous Septum

A recent systematic review and meta-analysis has confirmed what has been reported in multiple studies; namely that the length of the MS was inversely related to the risk of PPI [43]. Although some have reported that depth of implantation and radial force of the valve bioprosthesis is more predictive of post-TAVI conduction block [24], the weight of evidence still suggests that it is important to consider MS length before the TAVI procedure. Indeed, a single-center prospective cohort study found that a short MS length of less than 8 mm (7.69 mm being the optimal cutoff in terms of negative and positive predictive values) was strongly associated with the need for post-TAVI PPI [48]. This makes sense as the length of the MS can be considered a proxy measure of the depth below the aortic valve annulus where the conduction system crosses over to the left side of the heart [33].

#### 4.2.2. Distribution of Calcification and LVOT/Annulus Size and Shape

CT characterization studies have suggested other anatomic predictors of post-TAVI PPI such as a small LVOT diameter, the calcium volume below the NCC device-landing zone, left coronary cusp (LCC) calcification, or the volume of calcium in the LVOT below the left and right coronary cusps (RCC) [4,33,49,50,51]. However, some results have been conflicting, and severe LVOT calcification was an exclusion criterion in many TAVI trials [52]. Interestingly, ex vivo simulations of TAVI using a 3D-printed silicone annulus demonstrated that, in the case of LCC calcification, there was an off-center shift of the valvuloplasty balloon towards the commissure between the RCC and the NCC [50]. In a patient with a pre-existing RBBB, this situation would very likely lead to the need for PPI [50].

Although only 132 patients were studied, a recent report suggests that, in patients with particularly large annuli who had the self-expandable 34 mm Evolut R implanted, the strongest anatomic PPI predictor was the eccentricity index of the LVOT. This anatomic feature (and RBBB) correlated more strongly in terms of the prediction of PPI in this patient population than MS length [53].

### 4.3. Pre-Intervention EKG Predictors

As noted above, a pre-existing RBBB combined with a new, post-TAVI LBBB often results in the need for PPI [17]. In keeping with this, many studies have found that a pre-existing RBBB is a strong EKG predictor for the need for PPI after TAVI [35,43,49,51]. Even when controlling for the fact that patients have received different generations of valves and different types of valves, baseline RBBB remained a strong predictor of PPI. Baseline RBBB was associated with an increased PPI risk in patients who went on to receive first-generation valves (OR 4.68, 95% CI 3.53–5.83), second-generation valves (OR 3.30, 95% CI 2.03–4.59) self-expandable valves (OR 3.83, 95% CI 2.27–5.39), and balloon-expandable valves (OR 5.03, 95% CI 3.84–6.23) [54].

Other baseline atrioventricular conduction abnormalities have also been reported in two different meta-analyses to predict the need for PPI after TAVI. These baseline atrioventricular conduction blocks include first-degree AVB, LAHB, and Mobitz type-1 second-degree heart block [35,43].

Of note, baseline first-degree AVB and RBBB have also recently been reported to be associated with delayed high-grade AVB requiring PPI [55].

## 5. Procedural/Peri-Procedural Predictors

Patient-related predictors cannot be modified to decrease PPI risk. However, sometimes device choices can minimize this risk. Additionally, during the course of a TAVI and in the immediate post-TAVI period, there are procedural choices and peri-procedural observations that can further help predict the ultimate need for PPI.

- Valve choice

When interpreting the available literature on valve choice and PPI risk, the situation is ambiguous, as some studies mix different generations of valves or different types of valves. First-generation/early-generation valves include Medtronic CoreValve, Edwards Sapien, and Edwards Sapien XT. Second-generation/newer valves are generally considered to include: Edwards Sapien 3, Sapien 3 Ultra, Meril Life Sciences Myval, Medtronic CoreValve Evolute R, Medtronic Evolute PRO, Medtronic Evolute PRO+, Boston Scientific ACURATE *neo*, ACURATE *neo2*, Abbot Portico, Abbott Navilor, NVT ALLEGRA, Jenavalve Trilogy (which can treat both aortic regurgitation and aortic stenosis), and the now discontinued Boston Scientific Lotus [54]. When referring to TAVI valve type, they are generally classified according to how the valve frame is expanded: balloon-expandable (Edwards Sapien, Edwards Sapien XT, Edwards Sapien 3, Edwards Sapien 3 Ultra, and Meril Life Sciences Myval), self-expandable (Medtronic CoreValve, Medtronic CoreValve Evolut R, Medtronic Evolut Pro, Medtronic Evolut Pro+, Abbott Portico, Abbott Navitor, Boston Scientific ACURATE *neo*, Boston Scientific ACURATE *neo2*, NVT ALLEGRA, and Jenavalve Trilogy), and mechanical-expandable (Boston Scientific Lotus) ([54], and company websites as of March 2023). A summary of these commonly used valves in the US and/or EU, according to “generational” categories, can be found in Table 1.

Of these valves, the mechanical-expandable LOTUS Edge valve is no longer available. The FDA posted in January 2021 that Boston Scientific recalled and discontinued the „LOTUS Edge™ Aortic Valve System” [56]. The Lotus valve used controlled mechanical expansion and was fully retrievable and capable of recapturing and repositioning. It decreased the risk of paravalvular leaks because it sealed over irregular surfaces well, particularly as seen with heavily calcified LVOTs and bicuspid valves; however, it had a high rate of new LBBB and associated PPI [57,58]. One small study reported a new LBBB in 64.4% of patients and a PPI rate of 16.9% [58]. Thus, this valve, despite some positive features, is not a valve choice to be included in contemporary decision-making processes.

In a recent, well-done systematic review and meta-analysis, the first- and second-generation self-expandable valves (Medtronic CoreValve, Medtronic CoreValve Evolute R, Medtronic CoreValve Evolute Pro, and Abbot Portico valve) were significantly associated with PPI in the overall population (OR 2.99, 95% CI 1.39–4.59), and the pooled incidence rate of PPI for the self-expandable valves was 25% (95% CI 19–31%) [54]. Another systematic review and meta-analysis found a 1.93 and 2.8 times higher rate of PPM implantation with self-expanding and mechanically expandable prostheses, respectively, compared with balloon-expandable valves because self-expanding and mechanically expandable valves are often associated with deeper implantation into the aortic annulus, more tissue edema, and sustained pressure on the atrioventricular conduction system [43]. It was noted that such problems may be lessened or delayed with balloon-expandable valves because of the intermittent nature of expansion and minimized tissue impingement; although, second-generation balloon-expandable valves with an outer skirt (e.g., Sapien 3 Ultra) for paravalvular leak reduction might likewise increase PPI risk [13,43].

Recent progress in self-expandable valve design may be changing this picture. The Symetis ACURATE neo prosthesis is a self-expanding valve that uses a „2-step top-down“ deployment and has upper crowns that provide supra-annular anchoring and capping of the native leaflets, stabilization arches that aid in axial self-alignment, and a pericardial skirt to seal potential paravalvular leaks [59]. The one-year outcomes of the SAVI-TF (Symetis ACURATE neo Valve Implantation Using Transfemoral Access) registry reported favorable clinical outcomes and low PPI rates (9.9%, 95% CI 8.1–11.8%) [59]. ACURATE neo also performed well in terms of rate of PPI in a prospective comparison with CoreValve and Sapien XT: Accurate Neo 6% vs. Corevalve 25% vs. Sapien XT 11%; *p* = 0.013. This is thought to be due to the reduced generation of radial forces and supra-annular positioning [14]. Most recently, a reported finding of a subanalysis of SCOPE2 was that the rate of PPI with the ACCURATE neo was lower than that found with CoreValve Evolute (12.3% vs. 21.0%, respectively, *p* = 0.004) [60].

Of note, larger valves (29 mm and larger) in general are associated with an increased risk of PPI [43]. This is rather difficult to interpret as men generally need larger valves, and males have a higher risk of PPI. Rather than becoming stuck in viewing this as a “chicken or the egg” situation, perhaps one should just recognize both the male sex and large valve size as risk factors for PPI.

Finally, self-expanding valves (and new LBBB) were also associated with the delayed onset of high-grade AVB requiring PPI [55].

In Table 2 there is a comparison of the most relevant, recent clinical trials that looked at valve choice, patient risk as determined by the Society of Thoracic Surgeons (STS) risk score, mortality, risk of PPI, stroke, vascular complications, and procedural effectiveness as noted by gradient and paravalvular leak.

- Implantation depth

During TAVI, it has been found that the depth of valve prosthesis implantation is directly related to the risk of PPI [4,43]. Indeed, in consecutive patients receiving self-expandable valves during TAVI, it was found that the best predictor of PPI was the difference between MS length and implant depth [4,61]. Later, the anatomically guided MInimizing Depth According to the membranous Septum (MIDAS), which targets a prerelease depth in relation to the NCC of less than the length of the MS, was shown to decrease the rate of PPI with self-expanding valves from 9.7% to 3.0% (*p* =0.035), and the rate of new LBBB from 25.8% to 9% (*p* < 0.001) [62]. Thus, pre-procedural observation of MS length and procedural implantation depth go together as predictors of PPI, and information regarding these factors can be used to decrease PPI rates.

- Post-dilatation

Although balloon post-dilatation in TAVI can help decrease paravalvular leaks, there is also evidence that valvular balloon post-dilation after valve implantation independently predicts post-TAVI PPI (OR 9.21, 95% CI 5.46–15.54, *p* < 0.0001 [47].

- EKG changes

If no conduction abnormalities are present on pre-procedure and post-TAVI EKGs, then the rate of development of high-grade AVB has been found to be very low (1% at 24 h post-procedure) [55]. In contrast, certain EKG changes during the TAVI procedure or in the immediate post-TAVI period require extended monitoring to see if they are transient and, often, end up being predictive of PPI. EKG changes of concern include new-onset LBBB and/or increase in PR or QRS duration ≥20 ms, the development of transient or persistent complete heart block, and intraprocedural AVB regardless of valve type [4,22,43].

In particular, new-onset, persistent LBBB is feared to progress to high-grade AVB and necessary PPI. A systematic review and meta-analysis that examined the clinical impact at one-year post-TAVI of new, persistent LBBB found that it conferred an increased risk of PPI (RR 1.89, 95% CI 1.58–2.27; *p* <  0.001) [63]. Along the same lines, it has been reported that of TAVI patients who developed new, persistent LBBB and had ambulatory monitoring, 14% progressed to second or third-degree AVB within 30 days [18].

## 6. Risk Score

It would be useful to have a risk score or predictive model for PPI post-TAVI as it would help not only the Heart Team when considering the best approach for low-risk patients, but also would help the patients make more informed decisions in conjunction with their physicians. Likewise, it could aid in directing the choice of the prosthetic valve and the length and nature of post-TAVI monitoring.

To the best of our knowledge, a good predictive model that has been tested in multiple centers in large patient populations has not yet been published. However, Maeno, et al. reported the development of a model with a high negative predictive value in a single-center study using a balloon-expandable valve. The three pre-procedural independent predictors in this model were baseline RBBB, MS length, and NCC device-landing zone calcium volume [49]. After the addition of the procedural factor of device depth to the model (more specifically, the parameter was the difference between implantation depth and MS length), combined with RBBB and NCC device-landing zone calcium volume, yielded a c-statistic of 0.92, a sensitivity of 94.3%, a specificity of 83.8%, and a negative predictive value of 98.8% [49]. Further development of a risk score or predictive model remains a worthwhile goal.

## 7. Pre-Procedural Planning and Procedural Techniques to Optimize TAVI and Limit PPI

### 7.1. Valve Sizing

Careful pre-procedural planning for TAVI is centered today upon careful multi-detector computed tomography (MDCT) imaging with a TAVI protocol [64]. Attention is particularly paid to angulation of the aorta, ascending aorta diameter, aortic annulus diameters, aortic annulus area and perimeter, sinus of Valsalva dimensions, the height of the coronary arteries, dimensions of the sinotubular junction, LVOT diameters and perimeter, and distribution of calcification [64,65]. The aortic annulus dimensions are critical for selecting the best prosthetic valve size, with the perimeter guiding the sizing of self-expandable valves and the annulus area guiding the sizing of balloon-expandable valves. Both excessive oversizing and undersizing are to be avoided, as excessive oversizing of the valve can cause an increase in conduction blocks and undersizing can increase the risk of paravalvular leaks [64].

### 7.2. Coronary Ischemia and PPI

Rarely, during TAVI the prosthetic valve can cause acute ischemia, either by direct obstruction of a coronary artery or by indirect sequestering of the sinus of Valsalva at the sinotubular junction [66,67]. Lower height of the coronary ostia (<12 mm) and narrow sinus of Valsalva (<30 mm) increase the risk of this dangerous complication [68]. Additionally, the diameter and height of the sinotubular junction need to be carefully measured since when the sinotubular junction height is low, the balloon may injure it when a balloon-expandable valve is used. Furthermore, there can be sinus sequestration and coronary ischemia when the sinotubular junction is low and narrow in the setting of using a self-expandable valve or when dealing with valve-in-valve scenarios [64]. At times, the operator may need to make valve choices that may increase the risk of PPI but decrease the risk of ischemia, but pre-planning aids in optimizing this difficult situation. Recent reports have also suggested that patient-specific 3D models can help in pre-procedural planning for such challenging cases [69]. Likewise, intentional leaflet laceration with the BASILICA technique to prevent coronary artery obstruction might prove useful [66].

### 7.3. Implantation Height and MDCT Analysis, MIDAS Approach, Cusp-Overlap Technique, C-Arm Angulation

During the TAVI procedure, if careful attention is paid to implantation height (relative to MS length) and if the cusp-overlap technique is used with self-expanding valves, then the risk of PPI can be decreased. A higher implantation depth, particularly in patients with calcification under the NCC or with tapering of the LVOT below the aortic annulus, helps minimize damage to the AV conduction pathway and impingement of the MS during valve deployment [4]. As alluded to above, with self-expanding valves, the MIDAS approach described by Jilaihawi and colleagues at New York University can help to decrease the incidence of PPI because it is based upon implanting the valve at a depth that is less than the intraventricular MS length. Using a dedicated CT protocol with contrast and with retrospective EKG gating, various measurements are made including annular and LVOT size in mid-systole, calcium leaflet calcification using the J-score from the contrast scans, and, importantly, the MS is measured by determining the thinnest region of the interventricular septum on the perpendicular annular plane/axial image (generally lined up with the tricuspid annulus). On the corresponding stretched vessel image, the perpendicular vertical distance from the annular plane to the vertex of the muscular septum is measured. Prior to release, the prosthetic valve is positioned, relative to the non-coronary cusp, at a depth of less than the length of the MS [62].

**Figure 2:**
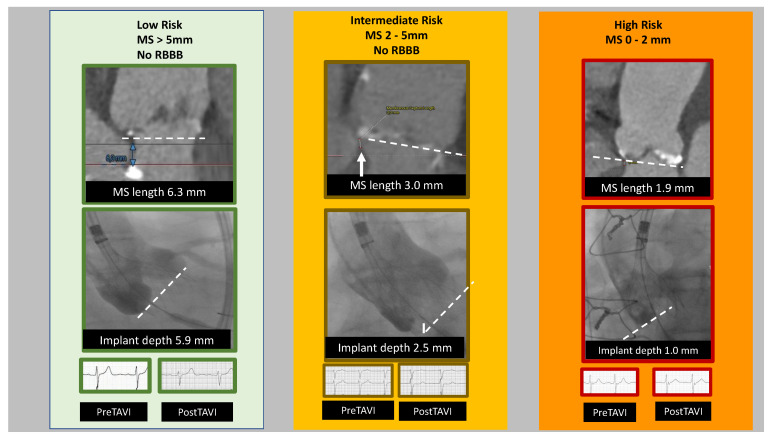
Membranous septal length and impact of implantation depth: A “new” anatomical concept. MS = Membranous Septum; RBBB = Right Bundle Branch Block.

An elegant retrospective study by Hokken and colleagues demonstrated that a careful MDCT analysis during pre-procedural planning for the MIDAS approach was vital to estimating and mitigating the risk of conduction disturbances. A software program (3mensio Structural Heart, Pie Medical Imaging, Maastricht, The Netherlands) was used to derive reconstructions from the EKG-gated, contrast scan in end-systole. In addition to the usual assessment of aortic root calcification, dimensions, and arterial access, the MS length was measured with the cursor placed on the intersection of the NCC and RCC, while in the perpendicular coplanar view. In this view, the MS was defined as the thinnest part of the interventricular septum between the LVOT and right atrium from the NCC nadir to the tip of the muscular interventricular septum, which is often delimited by the hinge point of the tricuspid valve’s septal leaflet. These investigators found three MS length-based “phenotypes” were associated with different risks for PPI post-TAVI. The high-risk phenotype had MS length <3 mm, the intermediate risk had an MS length of 3–6 mm, and the low-risk phenotype had MS >6 mm [70]. Figure 2 illustrates this interplay between implantation depth and MS length.

**Figure 3:**
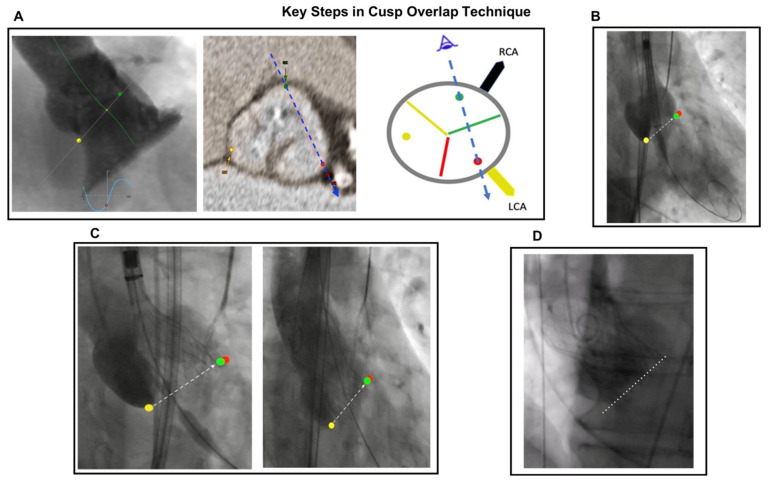
Key Steps in Cusp-Overlap Technique. (**A**) Step 1: a reconstructed computed tomography angiography overlay of the cusp-overlap view. (**B**) Step 2: a fluoroscopic view in RCC-LCC cusp overlap with Safari2 Wire appropriate in Left Ventricle and Implantation Starting Position. (**C**) Step 3: a fluoroscopic image demonstrates a 3 mm depth in the cusp-overlap view after full annular contact below the non-coronary cusp. (**D**) Step 4: a final aortography performed in the cusp-overlap view. Yellow color: Non Coronary Cusp (NCC); green color: Right Coronary Cusp (RCC); red color: Left Coronary Cusp (LCC); blue color: angle view, Dashed line: Annulus marker.

With self-expanding valves, the cusp-overlap technique also helps decrease the risk of PPI by overlapping the right coronary cusp and left coronary cusp and consequently isolating the NCC [71,72]. A coplanar view, typically the right anterior oblique/caudal projection, is found which allows for this, and the associated fluoroscopic angulation is called the cusp-overlap angulation [72]. This projection is valuable as it: minimizes parallax of the delivery catheter and allows it to be centered across the aortic valve, permits prosthetic valve deployment in a coplanar view, gives the en-face view of the NCC that allows for implantation of the prosthesis at a higher depth without device embolization, and improves visualization of the LVOT and MS during valve deployment [72,73]. To have good positioning of the valve prosthesis and alignment of the cusps, the correct angulations of the C-arm also need to be determined pre-procedurally by analysis of the MDCT data to avoid parallax [74,75]. A control view in the three-cusp annular plane is viewed before the final release. Figure 3 demonstrates the key steps in the cusp-overlap implantation technique. (Figure adapted from Lauten, PCR Online February 2022) The cusp-overlap technique may need to be adapted in patients who are obese, as the RAO, caudal view may yield images of reduced fluoroscopic quality. In such cases, our group uses a 3-cusp view or overlaps the NCC and the RCC in an LAO cranial view. (Lauten, PCR Online February 2022) Fortunately, most second-generation self-expandable TAVI platforms (e.g., Evolut valves/ACURATE/Navitor) allow for coronary artery cannulation post-TAVI when the cusp-overlap technique is used. (Lauten, PCR Online February 2022).

## 8. Post-Procedural Monitoring

**Figure 4:**
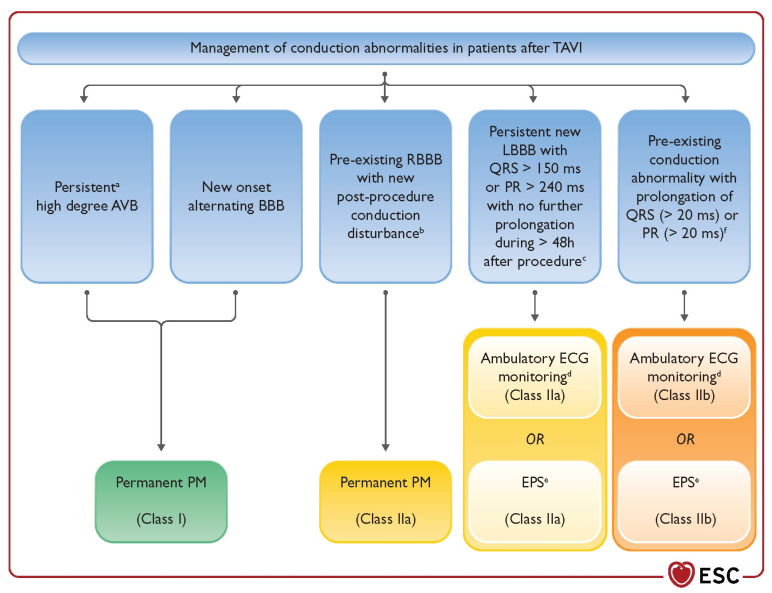
Management of conduction abnormalities after transcatheter aortic valve implantation. AF = atrial fibrillation; AV = atrioventricular; AVB = atrioventricular block; BBB = bundle branch block; ECG = electrocardiogram; EPS = electrophysiology study; HV = His–ventricular interval; LBBB = left bundle branch block; LVEF = left ventricular ejection fraction; PM = pacemaker; QRS = Q, R, and S waves; RBBB = right bundle branch block; TAVI = transcatheter aortic valve implantation. ^a^ 24–48 h post-procedure. ^b^ Transient high-degree AVB, PR prolongation, or axis change. ^c^ High-risk parameters for high-degree AV block in patients with new-onset LBBB include: AF, prolonged PR interval, and LVEF <40%. ^d^ Ambulatory continuous ECG monitoring for 7 − 30 days. ^e^ EPS with HV ≥70 ms may be considered positive for permanent pacing. ^f^ With no further prolongation of QRS or PR during 48-h observation. (Taken from [76]).

Guidelines and recommendations regarding PPI, telemetry, and ambulatory monitoring of patients post-TAVI have been evolving. A summary of the most recent ESC guidelines on pacing after AVI is in Figure 4 [76]. Unfortunately, some situations remain ambiguous, with it being particularly difficult to anticipate the clinical course of those without a pre-existing RBBB who develop an LBBB or an increase in PR or QRS interval of 20 ms or more post-TAVI, as the LBBB will frequently recover [4].

Just prior to the TAVI, a transvenous pacing wire is implanted as rapid ventricular pacing aids in valve implantation, and, in patients at high risk of conduction blocks or those who had procedural conduction blocks, it should remain [4]. If an intraprocedural complete or high-grade AVB persists for 24–48 h post-TAVI, then PPI is recommended according to 2021 ESC Guidelines. If there are new alternating bundle branch blocks, PPI is indicated [76]. Conversely, if patients have normal sinus rhythm and no new conduction blocks post-TAVI, then this temporary pacing wire and sheath can be removed, although telemetry for another 24 h and a follow-up EKG 24 h later is the standard procedure [4]. Likewise, if patients have pre-existing LBBB or first-degree AVB, then they can also have the temporary pacing wire removed and kept under telemetry for 24 h with a follow-up EKG 24 h later, so long as the PR and QRS intervals do not increase post-TAVI [4].

Patients without a pre-existing RBBB who develop a new LBBB or an increase in PR or QRS interval of ≥20 ms should have transvenous pacing for at least 24 h, continuous telemetry, and daily EKGs during their hospital stay [4]. If there is a new LBBB with QRS >150 ms or PR >240 ms (but no further prolongations of the intervals for more than 48 h post-TAVI, then ambulatory EKG monitoring or an electrophysiology study (performed at least three days post-TAVI and after conduction abnormalities are stable) could be considered per the ESC 2021 Guidelines [76]. Similarly, ambulatory monitoring or electrophysiology study may be considered when any pre-existing conduction abnormality is present and the QRS or PR intervals increase by more than 20 ms [76]. A recent small study reported that the temporary pacemaker wire to measure the His-ventricular (HV) interval in patients with post-TAVI LBBB could identify those patients who would not develop a delayed high-grade AVB, based upon an HV cutoff of 55 ms (90% negative predictive value) [77]. This could prove to be an easy way to identify earlier those at risk of delayed high-grade AVB and avoid unnecessary PPI in others.

The MARE Study found a high incidence of arrhythmic events during one year of follow-up with an implantable cardiac monitor in patients who had new and persistent LBBB post-TAVI, with PPI being necessary for almost half of them [32]. A smaller, single-center study found that post-TAVI patients with RBBB or new LBBB benefited from 30 days of continuous ambulatory monitoring due to the late development of AVB that ultimately required PPI [18].

As has been noted, patients with pre-existing RBBB, are at increased risk of developing high-grade AVB. Thus, even if their PR or QRS intervals remain stable, the transvenous pacemaker should remain for a minimum of 24 h and the patients monitored by telemetry. Their increased risk of developing high-grade AVB is greatest in the first week post-TAVI [4].

## 9. Discussion

The possible need for PPI post-TAVI remains a problem that needs to be discussed with patients, particularly lower-risk patients, prior to their procedure. As the use of TAVI expands, it is necessary to identify the most important predictors of PPI and to address any which are modifiable. Most patient-associated risks, such as pre-existing RBBB, short MS, male sex, age, small LVOT or eccentric LVOT with large annuli, and increased calcium volume below the non-coronary cusp, cannot be changed per se; however, they can influence pre-TAVI planning by the Heart Team [35,43,47,49,50,51,54]. Critical to this planning is MDCT imaging with precise analyses of measurements. This is necessary for techniques such as the MIDAS approach and cusp overlap, both of which can help reduce the risk of impingement of the MS and damage to the conduction system [62,72]. Good imaging likewise aids in proper valve sizing, as oversizing may lead to conduction blocks and undersizing may lead to paravalvular leaks [64]. Additionally, the procedure and valve choice can be adjusted when MDCT image analysis reveals that the patient has low coronary ostia and/or a narrow sinus of Valsalva, which increases the risk of the rare but dangerous complication of coronary ischemia; although, this may sometimes unfortunately mean that a possible iatrogenic injury to the conduction system has to be accepted over iatrogenically induced coronary ischemia [5,68].

Transient conduction blocks post-TAVI are common, and institutions should standardize telemetry and EKG monitoring post-procedure to distinguish between those blocks which are temporary from those which are permanent or advance to high-grade blocks requiring PPI [76]. Ambulatory, post-discharge monitoring may be needed not only for this but also for late high-grade blocks that may develop after discharge [17,39,40,41,55]. With such monitoring, one can hope to achieve a good balance so that both unnecessary, early PPI can be avoided as well as late cardiac syncope or death from post-discharge high-grade blocks.

Not discussed here is the risk of PPI found with valve-in-valve or repeat TAVI procedures. These topics are relatively new and deserving of extensive, separate treatment. Briefly, retrospective analyses have found that patients with degenerated, biological aortic prosthetic valves who underwent TAVI (valve-in-valve) procedures did not have an increased risk of PPI than the patients who underwent repeat surgical aortic valve replacement [78,79]. The CENTER study did not find a higher rate of PPI in valve-in-valve TAVI as compared to the native valve TAVI [80]. As TAVI is used more often now in lower-risk and younger patients, the need for re-do TAVI will increase. Data from large studies looking at this problem is lacking but will be needed in the future both to better understand the risk of PPI and the risk of coronary obstruction. A recent systematic review suggested that the rate of new PPI in redo-TAVI cases was 8.7% [81].

Also not discussed here is the situation with stenotic bicuspid aortic valves. Patients with bicuspid aortic valves are not homogenous in their valvular disorder and are often usually other difficult anatomic features for TAVI. However, results from the PARTNER 3 Bicuspid Registry seem to indicate that the rate of new PPI after TAVI for stenotic bicuspid valves is not different from that seen with stenotic tricuspid aortic valves [82].

## 10. Conclusions

Conduction disturbances post-TAVI remain a concerning complication. Recognizing a patient’s specific risk factors, careful MDCT imaging as part of pre-procedural planning, use of appropriate sized valves and techniques (MIDAS approach, cusp-overlap technique), and monitoring by telemetry, EKG, and even ambulatory device monitoring are all required to reduce the risk of both unnecessary early PPI and post-discharge cardiac emergencies secondary to late high-grade conduction blocks.

This is critical as post-TAVI PPI is associated with a higher risk of all-cause mortality and rehospitalization for heart failure. In general, this risk of PPI is higher with self-expanding valves than with balloon-expandable valves. Device positioning and implantation depth are important determinants of conduction disturbances after TAVI, with higher device implantation resulting in lower rates of PPI. Importantly, the interventricular MS is an anatomical landmark seen on the MDCT scan, which effectively functions as a surrogate for the distance between the native aortic annulus and the atrioventricular conduction system. The greater the difference between device implantation height relative to MS length (i.e., the difference between the MS length minus the device implantation height), the less the risk of conduction disturbances or PPI post-TAVI. 

Since a shorter MS length is associated with a higher risk of conduction disturbances resulting in PPI, we should include MS length measurement by MDCT in pre-TAVI planning and try to establish optimal implantation depth values before the procedure.

## 11. Future Directions

Currently, there is no pharmacological therapy for aortic valve stenosis, but it would be a major step forward for valvular heart disease if an effective one were found. Attenuation of valve calcification may delay or prevent the need for TAVI or surgical aortic valve replacement. A first-in-class drug, INS-3001, is a functionalized *myo*-inositol phosphate that may prove to be an inhibitor of pathological soft tissue calcification, especially vascular tissue and the aortic valve. However, its development is still at a very early stage, as a Phase I Clinical Trial is yet to be completed [83] 

### 11.1. Lipoprotein (a) [Lp(a)] and Aortic Valve Stenosis

High Lp(a) is associated with both microcalcification and macrocalcification of the aortic valve [84] especially in relatively young healthy individuals (45–54 years), in whom risk is increased three-fold at an Lp(a) > 80th percentile vs. lower levels (15.8% vs. 4.3%, respectively) [85]. High Lp(a) may also promote faster progression of aortic valve stenosis, culminating in earlier aortic valve replacement or death [84]. Randomized controlled trials lowering Lp(a) in aortic valve stenosis and its progression are needed.

### 11.2. Risk Score for PPI after TAVI

A more immediately achievable goal would be the development of an easy-to-use risk score to evaluate a patient’s risk of PPI post-TAVI. As noted above, this would be valuable not only when the Heart Team is planning a patient’s procedure, but also when discussing risks with the patient while obtaining informed consent. Maeno and colleagues have produced what may be a good prototype for a risk score, but this needs to be confirmed in large multi-center studies [49].

## Figures and Tables

**Table 1:** Most Common Valve Types Used in the USA and/or EU for TAVI.

Valve Type				
		Balloon- Expandable	Self-Expandable	Mechanical-Expandable
Generation	First/Early	Edwards Sapien Edwards Sapien XT	Medtronic CoreValve	
	Second/Newer	Edwards Sapien 3 Edwards Sapien 3 Ultra Meril Life Sciences Myval	Medtronic CoreValve Evolut R Medtronic Evolut PRO Medtronic Evolut PRO+ Abbott Portico Abbott Navitor Boston Scientific ACURATE *neo* Boston Scientific ACURATE *neo2* NVT ALLEGRAJenavalve Trilogy	Boston Scientific Lotus

**Table 2:** Clinical trials evaluating common valve options.

Company and Valve	Clinical Study Name	STS	PTS	All Cause Mortality 30 Days	All Cause Mortality 1 Year	Disabling Stroke 30 Days	Pacemaker Rate 30 Days	Moderate & Severe PVL 30 Days	Gradient 30 Days (mmHg)	Vascular Complications 30 Days
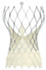 MEDTRONIC EVOLUTR/EVOLUTPRO	EvolutR CE	7.0	60	0.0%	5.7%	0.0%	11.7%	3.4%	8.1	8.3%
	SURTAVI	4.1	275	0.0%	5.5%	0.4%	16.1%	1.1%	8.9	3.6%
	Forward	5.5	1038	3.94%	8.9%	1.7%	17.5%	1.9%	8.5	5.5%
	EvolutPro US	6.4	60	1.7%	-	1.7%	11.25%	0.0%	6.4	10.0%
	NEOPRO	5.3	258	1.8%	-	2.5%	13.26%	5.7%	7.5	3.5%
	Solve	7.7	219	2.6%	17.6%	0.5%	22.98%	1.9%	-	-
	ForwardPro	4.7	629	3.2%	9.7%	2.5%	18.6%	1.8%	7.0	2.4%
	EvolutLowRisk	1.9	729	0.5%	2.4%	0.5%	17.4%	3.5%	8.4	3.8%
	Scope II	4.5	380	-	9.0%	-	16.04%	2.9%	-	-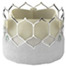 EDWARDS SAPIEN 3	Sapien 3 CE	7.5	95	2.5%	8.4%	0.0%	12.5%	2.6%	10.7	5.3%
	PARTNER 2 S3	8.4	491	3.5%	10.7%	2.1%	13.24%	2.9%	11.111.3	5.5%
	Source 3	4.8	1065	3.9%	11.2%	0.5%	12.5%	3.1%	11.0	4.1%
	Scope 1	3.4	357	0.8%	-	5.0%	10–32%	2.8%	11.5	5.5%
	SOLVE	7.6	219	2.3%	17.0%	4.7%	19.23%	1.4%	-	-
	PARTNER 3	1.9	496	0.4%	8.5%	0.5%	6.8%	0.8%	0.8	2.2%
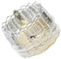 Boston Scientific Lotus Edge	REPRISE CE	6.5	250	4.0%	11.6%	2.5%	28.6%	0.6%	11.7	5.2%
	REPRISE II	6.7	248	2.5%	11.9%	2.0%	29.1%	-	12.0	7.0%
	RESPOND	6.0	996	2.2%	11.7%	2.3%	34.6%	0.3%	10.6	3.0%
	RESPOND Edge	3.2	100	0.0%	-	3.0%	22.6%	0.0%	10.1	4.0%
	Edge Euro Reg	3.2	256	2.4%	-	2.0%	30.8%	2.0%	11.2	2.1%
	REPRISE III	3.9	100	0.0%	6.0%	2.0%	20.1%	0.0%	14.0	6.0%
	Edge									
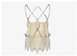 Boston Scientific Acurate Neo & Neo 2	Neo CE	6.8	82	3.4%	22.5%	2.2%	9.0%	4.9%	8.0	-
	SAVI TF Reg	6.0	924	1.5%	8.4%	1.9%	8.2%	4.1%	8.5	5.6%
	NEOPRO	5.0	1235	3.0%	-	2.096%	8.8%	5.2%	8.3	5.0%
	Scope I	3.7	372	2.6%	-	1.9%	11.5%	9.4%	7.0	7.0%
	Neo 2 CE	4.8	118	3.3%	12.2%	1.7%	15.05%	3.0%	7.9	3.3%
	PROGRESS PVL	6.0	490	2.2%	11.3%	2.4%	11.6%	5.0%	6.7	3.6%
	Scope II	4.0	390	-	13.0%	-	11.09%	9.6%	6.5	-
	ITAL – NEO					1.1%	11.0%			
	EARLY – NEO 2			1.3%		2.1%	6.0%	1.3%	9	
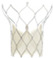	PORTICO CE	5.8	222	3.6%	-	3.2%	13.1%	5.5%	8.5	5.9%
	PORTICO 2	5.8	941	2.7%	12.1%	1.6%	18.7%	3.9%	8.6	5.5%
	PORTICO DIE	6.4	381	3.5%	14.3%	1.5%	13.4%	6.3%	8.4	9.0%
	FLEXNAV CE	3.3	150	0.6%	-	1.2%	15.4%	4.1%	7.1	5.0%
Abbott										
PORTICO										

## References

[1] De Torres-Alba F., Kaleschke G., Diller G.P., Vormbrock J., Orwat S., Radke R., Reinke F., Fischer D., Reinecke H., Baumgartner H. (2016). Changes in the Pacemaker Rate after Transition from Edwards SAPIEN XT to SAPIEN 3 Transcatheter Aortic Valve Implantation. JACC Cardiovasc. Interv..

[2] Van der Boon R.M.A., Houthuizen P., Urena P., Poels T.T., van Miegham N.M., Brueren G.R.G., Altintas S., Nuis R.J., Serruys P.W., van Garsse L.A.F.M. (2015). Trends in occurrence of new conduction abnormalities after transcatheter aortic valve implantation. Catheter. Cardiovasc. Interv..

[3] Husser O., Pellegrini C., Kessler T., Burgdorf C., Thaller H., Mayr N.P., Kasel A.M., Kastrati A., Schunkert H., Hengstenberg C. (2016). Predictors of Permanent Pacemaker Implantations and New-Onset Conduction Abnormalities with the SAPIEN3 Balloon-Expandable Transcatheter Heart Valve. JACC Cardiovasc. Interv..

[4] Lilly S.M., Deshmukh A.J., Epstein A.E., Ricciardi M.J., Shreenivas S., Velagapudi P., Wyman J.F. (2020). 2020 ACC expert consensus decision pathway on management of conduction disturbances in patients undergoing transcatheter aortic valve replacement. J. Am. Coll. Cardiol..

[5] Piazza N., de Jaegere P., Schultz C., Becker A.E., Serruys P.W., Anderson R.H. (2008). Anatomy of the Aortic Valvar Complex and Its Implications for Transcatheter Implantation of the Aortic Valve. Circ. Cardiovasc. Interv..

[6] Mack M.J., Leon M.B., Thourani V.H., Makkar R., Kodali S.K., Russo M., Kapadia S.R., Malaisrie S.C., Cohen D.J., Pibarot P. (2019). PARTNER 3 Investigators. Transcather aortic-valve replacement with a balloon-expandable valve in low-risk patients. N. Engl. J. Med..

[7] Popma J.J., Deeb G.M., Yakubov S.J., Mumtaz M., Gada H., O’Hair D., Bajwa T., Heiser J.C., Merhi W., Kleiman N.S. (2019). Evolut Low Risk Trial Investigators Transcatheter aortic-valve replacement with a self-expanding valve in low-risk patients. N. Engl. J. Med..

[8] Leon M.B., Mack M.J., Hahn R.T., Thourani V.H., Makkar R., Kodali S.K., Alu M.C., Madhavan M.V., Chau K.H., Russo M. (2021). PARTNER 3 Investigators, Outcomes 2 years after transcatheter aortic valve replacement in patients at low surgical risk. J. Am. Coll. Cardiol..

[9] Elder D.H.J., Lang C.C., Choy A. (2011). M Pacing-induced heart disease: Understanding the pathophysiology and improving outcomes. Expert Rev. Cardiovasc. Ther..

[10] Akerström F., Arais M.A., Pachón M., Jiménez-López J., Puchol A., Juliá-Calvo J. (2013). The importance of avoiding unnecessary right ventricular pacing in clinical practice. World J. Cardiol..

[11] Curtis A.B., Worley S.J., Adamson P.B., Chung E.S., Niazi I., Sherfesee L., Shinn T., Sutton M.S.J. (2013). Biventricular Pacing for Atrioventricular Block and Systolic Dysfunction. N. Engl. J. Med..

[12] Rück A., Saleh N., Glaser N. (2021). Outcomes Following Permanent Pacemaker Implantation after Transcatheter Aortic Valve Replacement. J. Am. Coll. Cardiol. Interv..

[13] Bisson A., Bodin A., Herbert J., Lacour T., Saint Etienne C., Pierre B., Clementy N., Deharo P., Babuty D., Fauchier L. (2020). Pacemaker Implantation after Balloon- or Self-Expandable Transcatheter Aortic Valve Replacement in Patients with Aortic Stenosis. J. Am. Heart Assoc..

[14] Huang H., Mansour M. (2020). Pacemaker Implantation after Transcatheter Aortic Valve Replacement: A Necessary Evil Perhaps but Are We Making Progress?. J. Am. Heart Assoc..

[15] Nazif T.M., Dizon J.M., Hahn R.T., Xu K., Babaliaros V., Douglas P.S., El-Chami M.F., Herrmann H., Mack M., Makkar R.R. (2015). Predictors and Clinical Outcomes of Permanent Pacemaker Implantation after Transcatheter Aortic Valve Replacement The PARTNER (Placement of AoRtic TraNscathetER Valves) Trial and Registry. J. Am. Coll. Cardiol. Interv..

[16] Poels T.T., Engels E.B., Kats S., Veenstra L., van Ommen V., Vernooy K., Maessen J.G., Prinzen F.W. (2021). Occurrence and Persistency of Conduction Disturbances during Transcatheter Aortic Valve Implantation. Medicina.

[17] Barbanti M., Baan J., Spence M.S., Iacovelli F., Martinelli G.L., Saia F., Bortone A.S., Van der Kley F., Muir D.F., Densem C.G. (2017). Feasibility and safety of early discharge after transfemoral transcatheter aortic valve implantation–rationale and design of the FAST-TAVI registry. BMC Cardiovasc. Disord..

[18] Tian Y., Padmanabhan D., McLeod C.J., Zhang P., Xiao P., Sandhu G.S., Greason K.L., Gulati R., Nkomo V., Rihal C.S. (2019). Utility of 30-Day Continuous Ambulatory Monitoring to Identify Patients with Delayed Occurrence of Atrioventricular Block after Transcatheter Aortic Valve Replacement. Circ. Cardiovasc. Interv..

[19] Bagur R., Rodés-Cabau J., Gurvitch R., Dumont É., Velianou J.L., Manazzoni J., Toggweiler S., Cheung A., Ye J., Natarajan M.K. (2012). Need for permanent pacemaker as a complication of transcatheter aortic valve implantation and surgical aortic valve replacement in elderly patients with severe aortic stenosis and similar baseline electrocardiographic findings. JACC Interv..

[20] Roten L., Stortecky S., Scarcia F., Kadner A., Tanner H., Delacrétaz E., Meier B., Windecker S., Carrel T., Wenaweser P. (2012). Atrioventricular Conduction after Transcatheter Aortic Valve Implantation and Surgical Aortic Valve Replacement. J. Cardiovasc. Electrophysiol..

[21] Kanjanauthai S., Bhasin K., Pirelli L., Kliger C. (2019). Conduction Abnormalities after Transcatheter Aortic Valve Replacement. US Cardiol. Rev..

[22] Mangieri A., Montalto C., Pagnesi M., Lanzillo G., Demir O., Testa L., Colombo A., Latib A. (2018). TAVI and Post Procedural Cardiac Conduction Abnormalities. Front. Cardiovasc. Med..

[23] Kawashima T., Sato F. (2014). Visualizing anatomical evidences on atrioventricular conduction system for TAVI. Int. J. Cardiol..

[24] Tretter J.T., Mori S., Anderson R.H., Taylor M.D., Ollberding N., Truong V., Choo J., Kereiakes D., Mazur W. (2019). Anatomical predictors of conduction damage after transcatheter implantation of the aortic valve. Open Heart.

[25] Lin S.-I., Miura M., Tagliari A.P., Lee Y.-H., Shirai S., Puri R., Maisano F., Taramasso M. (2020). Intraventricular Conduction Disturbances After Transcatheter Aortic Valve Implantation. Interv. Cardiol. Rev..

[26] Massoullié G., Bordachar P., Ellenbogen K.A., Souteyrand G., Jean F., Combaret N., Vorilhon C., Clerfond G., Farhat M., Ritter P. (2016). New-onset left bundle branch block induced by transcutaneous aortic valve implantation. Am. J. Cardiol..

[27] Hamandi M., Tabachnick D., Lanfear A.T., Baxter R., Shin K., Zingler B., Mack M.J., DiMaio J.M., Kindsvater S. (2020). Effect of new and persistent left bundle branch block after transcatheter aortic valve replacement on long-term need for pacemaker implantation. Proc. Baylor.Univ. Med. Cent..

[28] Jørgensen T.H., De Backer O., Gerds T.A., Bieliauskas G., Svendsen J.H., Søndergaard L.J. (2018). Immediate Post-Procedural 12-Lead Electrocardiography as Predictor of Late Conduction Defects after Transcatheter Aortic Valve Replacement. Am. Coll. Cardiol. Interv..

[29] Houthuizen P., van der Boon R.M.A., Urena M., Van Mieghem N., Brueren G.B.R., Poels T.T., Van Garsse L.A.F.M., Rodés-Cabau J., Prinzen F.W., de Jaegere P. (2014). Occurrence, fate and consequences of ventricular conduction abnormalities after transcatheter aortic valve implantation. EuroIntervention.

[30] Urena M., Mok M., Serra V., Dumont E., Nombela-Franco L., DeLarochellière R., Doyle D., Igual A., Larose E., Amat-Santos I. (2012). Predictive Factors and Long-Term Clinical Consequences of Persistent Left Bundle Branch Block Following Transcatheter Aortic Valve Implantation with a Balloon-Expandable Valve. J. Am. Coll. Cardiol..

[31] Nazif T.M., Chen S., George I., Dizon J.M., Hahn R.T., Crowley A., Alu M.C., Babaliaros V., Thourani V.H., Herrmann H.C. (2019). New-onset left bundle branch block after transcatheter aortic valve replacement is associated with adverse long-term clinical outcomes in intermediate-risk patients: An analysis from the PARTNER II trial. Eur. Heart J..

[32] Rodés-Cabau J., Urena M., Nombela-Franco L., Amat-Santos I., Kleiman N., Munoz-Garcia A., Atienza F., Serra V., Deyell M.W., Veiga-Fernandez G. (2018). Arrhythmic Burden as Determined by Ambulatory Continuous Cardiac Monitoring in Patients with New-Onset Persistent Left Bundle Branch Block following Transcatheter Aortic Valve Replacement the MARE Study. J. Am. Coll. Cardiol. Interv..

[33] Chen S., Chau K.T., Nazif T.M. (2020). The incidence and impact of cardiac conduction disturbances after transcatheter aortic valve replacement. Ann. Cardiothorac. Surg..

[34] Kostopoulou A., Karyofillis P., Livanis E., Thomopoulou S., Stefopoulos C., Doudoumis K., Theodorakis G., Voudris V. (2016). Permanent pacing after transcatheter aortic valve implantation of a CoreValve prosthesis as determined by electrocardiographic and electrophysiological predictors: A single-centre experience. Europace.

[35] Siontis G.C.M., Jüni P., Pilgrim T., Stortecky S., Büllesfeld L., Meier B., Wenaweser P., Windecker S. (2014). Predictors of Permanent Pacemaker Implantation in Patients with Severe Aortic Stenosis Undergoing TAVR: A Meta-Analysis. J. Am. Coll. Cardiol..

[36] Mazzella A.J., Sanders M., Yang H., Li Q., Vavalle J.P., Gehi A. (2020). Predicting need for pacemaker implantation early and late after transcatheter aortic valve implantation. Catheter. Cardiovasc. Interv..

[37] Mazzella A.J., Arora S., Hendrickson M.J., Sanders M., Vavalle J.P., Gehi A.K. (2021). Evaluation and Management of Heart Block after Transcatheter Aortic Valve Replacement. Card. Fail. Rev..

[38] Urena M., Webb J.G., Tamburino C., Muñoz-García A.J., Cheema A., Dager A.E., Serra V., Amat-Santos I.J., Barbanti M., Immè S. (2014). Permanent pacemaker implantation after transcatheter aortic valve implantation: Impact on late clinical outcomes and left ventricular function. Circulation.

[39] Mazzella A.J., Hendrickson M.J., Arora S., Sanders M., Li Q., Vavalle J.P., Gehi A.K. (2021). Shifting Trends in Timing of Pacemaker Implantation after Transcatheter Aortic Valve Replacement. JACC CV Interv..

[40] Rodés-Cabau J., Ellenbogen K.A., Krahn A.D., Latib A., Mack M., Mittal S., Muntané-Carol G., Nazif T.M., Sondergaard L., Urena M. (2019). Management of Conduction Disturbances Associated with Transcatheter Aortic Valve Replacement JACC Scientific Expert Panel. J. Am. Coll. Cardiol..

[41] Kalogeropoulos A.S., Redwood S.R., Allen C.J., Hurrell H., Chehab O., Rajani R., Prendergast B., Patterson T. (2022). A 20-year journey in transcatheter aortic valve implantation: Evolution to current eminence. Front. Cardiovasc. Med..

[42] Toggweiler S., Stortecky S., Holy E., Zuk K., Cuculi F., Nietlispach F., Sabti Z., Suciu R., Maier W., Jamshidi P. (2016). The Electrocardiogram after Transcatheter Aortic Valve Replacement Determines the Risk for Post-Procedural High-Degree AV Block and the Need for Telemetry Monitoring. J. Am. Coll. Cardiol. Interv..

[43] Ullah W., Zahid S., Zaidi S.R., Sarvepalli D., Haq S., Rommi S., Mukhtar M., Khan M.A., Gowda S.N., Ruggiero N. (2021). Predictors of Permanent Pacemaker Implantation in Patients Undergoing Transcatheter Aortic Valve Replacement–A Systematic Review and Meta-Analysis. J. Am. Heart Assoc..

[44] Elbaz-Greener G., Rahamim E., Ghosh Z.A., Carasso S., Yarkoni M., Radhakrishnan S., Wijeysundera H.C., Igor T., Planer D., Rozen G. (2022). Sex difference and outcome trends following transcatheter aortic valve replacement. Front. Cardiovasc. Med..

[45] Ravaux J.M., Van Kuijk S.M.J., Di Mauro M., Vernooy K., Bidar E., Van’t Hof A.W., Veenstra L., Kats S., Houterman S., Maessen J.G. (2022). Incidence and Predictors of Permanent Pacemaker Implantation after Transcatheter Aortic Valve Procedures: Data of The Netherlands Heart Registration (NHR). J. Clin. Med..

[46] Attinger-Toller A., Ferrari E., Tueller D., Templin C., Muller O., Nietlispach F., Toggweiler S., Noble S., Roffi M., Jeger R. (2021). Age-Related Outcomes after Transcatheter Aortic Valve Replacement Insights From the Swiss TAVI Registry. J. Am. Coll. Cardiol. Interv..

[47] Giustino G., Van der Boon R.M.A., Molina-Martin de Nicolas J., Dumonteil N., Chieffo A., de Jaegere P.P.T., Tchetche D., Marcheix B., Millischer D., Cassagneau R. (2016). Impact of permanent pacemaker on mortality after transcatheter aortic valve implantation: The PRAGMATIC (Pooled Rotterdam-Milan-Toulouse in Collaboration) Pacemaker substudy. EuroIntervention.

[48] Gama F., de Araújo Gonçalves P., Abecasis J., Ferreira A.M., Freitas P., Gonçalves M., Carvalho S., Oliveira A.F., Gabriel H.M., Brito J. (2022). Predictors of pacemaker implantation after TAVI in a registry including self, balloon and mechanical expandable valves. Int. J. Cardiovasc. Imaging.

[49] Maeno Y., Abramowitz Y., Kawamori H., Kazuno Y., Kubo S., Takahashi N., Mangat G., Okuyama K., Kashif M., Chakravarty T. (2017). A Highly Predictive Risk Model for Pacemaker Implantation after TAVR. J. Am. Coll. Cardiol. Imaging.

[50] Fujita B., Kütting M., Seiffert M., Scholtz S., Egron S., Prashovikj E., Börgermann J., Schäfer T., Scholtz W., Preuss R. (2016). Calcium distribution patterns of the aortic valve as a risk factor for the need of permanent pacemaker implantation after transcatheter aortic valve implantation. Eur. Heart J.-Cardiovasc. Imaging.

[51] Mauri V., Reimann A., Stern D., Scherner M., Kuhn E., Rudolph V., Rosenkranz S., Eghbalzadeh K., Friedrichs K., Wahlers T. (2016). Predictors of Permanent Pacemaker Implantation after Transcatheter Aortic Valve Replacement with the SAPIEN 3. J. Am. Coll. Cardiol. Interv..

[52] Farhan S., Stachel G., Desch S., Kurz T., Feistritzer H.-J., Hartung P., Eitel I., Nef H., Doerr O., Lauten A. (2022). Impact of moderate or severe left ventricular outflow tract calcification on clinical outcomes of patients with severe aortic stenosis undergoing transcatheter aortic valve implantation with self- and balloon-expandable valves: A post hoc analysis from the SOLVE-TAVI trial. EuroIntervention.

[53] Veulemans V., Frank D., Seoudy H., Wundram S., Piayda K., Maier O., Jung C., Polzin A., Frey N., Kelm M. (2020). New insights on potential permanent pacemaker predictors in TAVR using the largest self-expandable device. Cardiovasc. Diagn. Ther..

[54] Bruno F., D’Ascenzo F., Vaira M.P., Elia E., Omedè P., Kodali S., Barbanti M., Rodés-Cabau J., Husser O., Sossalla S. (2021). Predictors of pacemaker implantation after transcatheter aortic valve implantation according to kind of prosthesis and risk profile: A systematic review and contemporary meta-analysis. Eur. Heart J..

[55] El-Sabawi B., Welle G.A., Cha Y.-M., Espinosa R.E., Gulati R., Sandhu G.S., Greason K.L., Crestanello J.A., Friedman P.A., Munger T.M. (2021). Temporal Incidence and Predictors of High-Grade Atrioventricular Block after Transcatheter Aortic Valve Replacement. J. Am. Heart Assoc..

[56] https://www.fda.gov/safety/recalls-market-withdrawals-safety-alerts/boston-scientific-announces-lotus-edgetm-aortic-valve-system-voluntary-recall-and-product#recall-announcement.

[57] Solomonica A., Choudhury T., Bagur R. (2018). The mechanically expandable LOTUS Valve and LOTUS Edge transcatheter aortic valve systems. Expert Rev. Med. Devices.

[58] Medranda G.A., Rogers T., Case B.C., Shults C.C., Cohen J.E., Satler L.F., Ben-Dor I. (2021). Waksman R Single-Center Experience with the LOTUS Edge Transcatheter Heart Valve. Cardiovasc. Revascularization Med..

[59] Kim W.-K., Hengstenberg C., Hilker M., Kerber S., Schäfer U., Rudolph T., Linke A., Franz N., Kuntze T., Nef H. (2018). The SAVI-TF Registry 1-Year Outcomes of the European Post-Market Registry Using the ACURATE neo Transcatheter Heart Valve Under Real-World Conditions in 1000 Patients. Am. Coll. Cardiol. Interv..

[60] Pellegrini C., Garot P., Morice M.-C., Tamburino C., Bleiziffer S., Thiele H., Scholtz S., Schramm R., Cockburn J., Cunnington M. (2023). Permanent pacemaker implantation and left bundle branch block with self-expanding valves—A SCOPE 2 subanalysis. EuroIntervention.

[61] Hamdan A., Guetta V., Klempfner R., Konen E., Raanani E., Glikson M., Goitein O., Segev A., Barbash I., Fefer P. (2015). Inverse Relationship Between Membranous Septal Length and the Risk of Atrioventricular Block in Patients Undergoing Transcatheter Aortic Valve Implantation. J. Am. Coll. Cardiol. Interv..

[62] Jilaihawi H., Zhao Z., Du R., Staniloae C., Saric M., Neuburger P.J., Querijero M., Vainrib A., Hisamoto K., Ibrahim H. (2019). Jilaihawi Minimizing Permanent Pacemaker Following Repositionable Self-Expanding Transcatheter Aortic Valve Replacement. J. Am. Coll. Cardiol. Interv..

[63] Faroux L., Chen S., Muntané-Carol G., Regueiro A., Philippon F., Sondergaard L., Jørgensen T.H., Lopez-Aguilera J., Kodali S., Leon M. (2020). Clinical impact of conduction disturbances in transcatheter aortic valve replacement recipients: A systematic review and meta-analysis. Eur. Heart J..

[64] Saadi R.P., Tagliari A.P., Saadi E.K., Miglioranza M.H., Polanczyck C.A. (2022). Preoperative TAVR Planning: How to Do It. J. Clin Med..

[65] Blanke P., Euringer W., Baumann T., Reinöhl J., Schlensak C., Langer M., Pache G. (2010). Combined Assessment of Aortic Root Anatomy and Aortoiliac Vasculature with Dual-Source CT as a Screening Tool in Patients Evaluated for Transcatheter Aortic Valve Implantation. Am. J. Roentgenol..

[66] Westermann D., Ludwig S., Kalbacher D., Spink C., Linder M., Bhandra O.D., Nikorowitsch J., Waldschmidt L., Demal T., Voigtländer L. (2021). Prevention of coronary obstruction in patients at risk undergoing transcatheter aortic valve implantation: The Hamburg BASILICA experience. Clin. Res. Cardiol..

[67] Arai T., Lefèvre T., Hovasse T., Garot P., Benamer H., Unterseeh T., Roy A.K., Romano M., Hayashida K., Watanabe Y. (2017). Incidence and predictors of coronary obstruction following transcatheter aortic valve implantation in the real world. Catheter. Cardiovasc. Interv..

[68] Blanke P., Weir-McCall J.R., Achenbach S., Delgado V., Hausleiter J., Jilaihawi H., Marwan M., Nørgaard B.L., Piazza N., Schoenhagen P. (2019). Computed Tomography Imaging in the Context of Transcatheter Aortic Valve Implantation (TAVI)/Transcatheter Aortic Valve Replacement (TAVR) An Expert Consensus Document of the Society of Cardiovascular Computed Tomography. JACC Cardiovasc. Imaging.

[69] Xenofontos P., Zamani R., Akrami M. (2022). The application of 3D printing in preoperative planning for transcatheter aortic valve replacement: A systematic review. BioMed Eng. OnLine.

[70] Hokken T.W., van Wiechen M.P., Ooms J.F., El Azzouzi I., de Ronde M., Kardys I., Budde R., Daemen J., de Jaegere P.P., Van Mieghem N.M. (2021). Impact of Interventricular membranous septum length on pacemaker need with different Transcatheter aortic valve implantation systems. Int. J. Cardiol..

[71] Tang G.H.L., Zaid S., Michev I., Ahmad H., Kaple R., Undemir C., Cohen M., Lansman S.L. (2018). “Cusp-Overlap” View Simplifies Fluoroscopy-Guided Implantation of Self-Expanding Valve in Transcatheter Aortic Valve Replacement. JACC Cardiovasc. Interv..

[72] Sengupta A., Alexis S.L., Lee T., Zaid S., Krishnamoorthy P.M., Khera S., Lerakis S., Anastasius M., Dangas G.D., Sharma S.K. (2022). Cusp Overlap Technique: Should it become the Standard Implantation Technique for Self-Expanding Valves?. Curr. Cardiol. Rep..

[73] Doldi P.M., Stolz L., Escher F., Steffen J., Gmeiner J., Roden D., Linnemann M., Löw K., Deseive S., Stocker T.J. (2022). Transcatheter Aortic Valve Replacement with the Self-Expandable Core Valve Evolut Prosthesis Using the Cusp-Overlap vs. Tricusp-View. J. Clin. Med..

[74] Immè S., Attizzani G.F., Sgroi C., Barbanti M., Patané M., Capodanno D., Tamburino C. (2013). Pre-defining optimal C-arm position for TAVI with CT-scan using free software. EuroIntervention.

[75] Samim M., Stella P.R., Agostoni P., Kluin J., Ramjankhan F., Budde R.P.J., Sieswerda G., Algeri E., van Belle C., Elkalioubie A. (2013). Automated 3D Analysis of Pre-Procedural MDCT to Predict Annulus Plane Angulation and C-Arm Positioning. JACC Cardiovasc. Imaging.

[76] Glikson M., Nielsen J.C., Kronborg M.B., Michowitz Y., Auricchio A., Barbash I.M., Barrabés J.A., Boriani G., Braunschweig F., Brignole M. (2021). 2021 ESC Guidelines on cardiac pacing and cardiac resynchronization therapy: Developed by the Task Force on cardiac pacing and cardiac resynchronization therapy of the European Society of Cardiology (ESC) with the special contribution of the European Heart Rhythm Association (EHRA). Eur. Heart J..

[77] Knecht S., Schaer B., Reichlin T., Spies F., Madaffari A., Vischer A., Fahmi G., Jeger R., Kaiser C., Osswald S. (2020). Electrophysiology Testing to Stratify Patients with Left Bundle Branch Block after Transcatheter Aortic Valve Implantation. J. Am. Heart Assoc..

[78] van Steenberger G.J., van Straten B., Lam K.Y., van Veghel D., Dekker L., Tonino P.A. (2022). Report on outcomes of valve-in-valve transcatheter aortic valve implantation and redo surgical aortic valve replacement in the Netherlands. Neth. Heart J..

[79] Majmundar M., Doshi R., Kumar A., Johnston D., Brockett J., Kanaa’N A., Lahorra J.A., Svensson L.G., Krishnaswamy A., Reed G.W. (2022). Valve-in-valve transcatheter aortic valve implantation versus repeat surgical aortic valve replacement in patients with a failed aortic bioprosthesis. EuroIntervention.

[80] van Nieuwkerk A.C., Santos R.B., Fernandez-Nofrerias E., Tchétché D., de Brito F.S., Barbanti M., Kornowski R., Latib A., D’Onofrio A., Ribichini F. (2022). Outcomes in Valve-in-Valve Transcatheter Aortic Valve Implantation. Am. J. Cardiol..

[81] Gallo M., Fovino L.N., Blitzer D., Doulamis I.P., Guariento A., Salvador L., Tagliari A.P., Ferrari E. (2022). Transcatheter aortic valve replacement for structural degeneration of previously implanted transcatheter valves (TAVR-in-TAVR): A systematic review. Eur. J. Cardio-Thorac. Surg..

[82] Williams M.R., Jilaihawi H., Makkar R., O’Neill W.W., Guyton R., Malaisrie C., Brown D.L., Blanke P., Leipsic J.A., Pibarot P. (2022). The PARTNER 3 Bicuspid Registry for Transcatheter Aortic Valve Replacement in Low-Surgical-Risk Pati. J. Am. Coll. Cardiol. Interv..

[83] Shantl A.E., Verhulst A., Neven E., Behets G.J., D’Haese P.C., Maillard M., Mordasini D., Phan O., Burnier M., Spaggiari D. (2020). Inhibition of vascular calcification by inositol phosphates derivatized with ethylene glycol oligomers. Nat. Commun..

[84] Despres A.A., Perrot N., Poulin A., Tastet L., Shen M., Chen H.Y., Bourgeois R., Trottier M., Tessier M., Guimond J. (2019). Lipoprotein(a), Oxidized Phospholipids, and Aortic Valve Microcalcification Assessed by 18F-Sodium Fluoride Positron Emission Tomography and Computed Tomography. CJC Open.

[85] Kaiser Y., Singh S.S., Zheng K.H., Verbeek R., Kavousi M., Pinto S.J., Vernooij M.W., Sijbrands E.J.G., Boekholdt S.M., de Rijke Y.B. (2021). Lipoprotein(a) is robustly associated with aortic valve calcium. Heart.

